# Tunneling Nanotubes: Intimate Communication between Myeloid Cells

**DOI:** 10.3389/fimmu.2018.00043

**Published:** 2018-01-25

**Authors:** Maeva Dupont, Shanti Souriant, Geanncarlo Lugo-Villarino, Isabelle Maridonneau-Parini, Christel Vérollet

**Affiliations:** ^1^Institut de Pharmacologie et de Biologie Structurale, Université de Toulouse, CNRS, Université Toulouse III Paul Sabatier, Toulouse, France; ^2^Research Program “IM-TB/HIV” (1167), International Associated Laboratory (LIA), CNRS, Toulouse, France

**Keywords:** tunneling nanotubes, myeloid cells, innate immunity, pathogens, HIV-1

## Abstract

Tunneling nanotubes (TNT) are dynamic connections between cells, which represent a novel route for cell-to-cell communication. A growing body of evidence points TNT towards a role for intercellular exchanges of signals, molecules, organelles, and pathogens, involving them in a diverse array of functions. TNT form among several cell types, including neuronal cells, epithelial cells, and almost all immune cells. In myeloid cells (e.g., macrophages, dendritic cells, and osteoclasts), intercellular communication *via* TNT contributes to their differentiation and immune functions. Importantly, TNT enable myeloid cells to communicate with a targeted neighboring or distant cell, as well as with other cell types, therefore creating a complex variety of cellular exchanges. TNT also contribute to pathogen spread as they serve as “corridors” from a cell to another. Herein, we addressed the complexity of the definition and *in vitro* characterization of TNT in innate immune cells, the different processes involved in their formation, and their relevance *in vivo*. We also assess our current understanding of how TNT participate in immune surveillance and the spread of pathogens, with a particular interest for HIV-1. Overall, despite recent progress in this growing research field, we highlight that further investigation is needed to better unveil the role of TNT in both physiological and pathological conditions.

## Introduction

Tunneling nanotubes (TNT) represent a novel type of intercellular communication machinery, which differs from the secretion of signaling molecules and the signal transmission through gap or synaptic junctions between adjacent cells. Along with exosomes, TNT mediate long-range communication, independent of soluble factors. They are membranous structures displaying a remarkable capacity to communicate with selected neighbor or distant cells. There are recent reviews covering the broad biological role of TNT, which are able to form in multiple cell types (1–3). Here, our focus is exclusively on TNT formed by myeloid cells, including macrophages, osteoclasts, and dendritic cells (DC). Based on the nascent literature on TNT in these cells, we will discuss the definition of TNT, their mechanisms of formation, and their role in physiological and pathological contexts. We will also address the need of further investigation of these structures to better understand their functions and improve their potential as therapeutic targets in pathological conditions.

## Definition and Function of TNT

**Figure 1:**
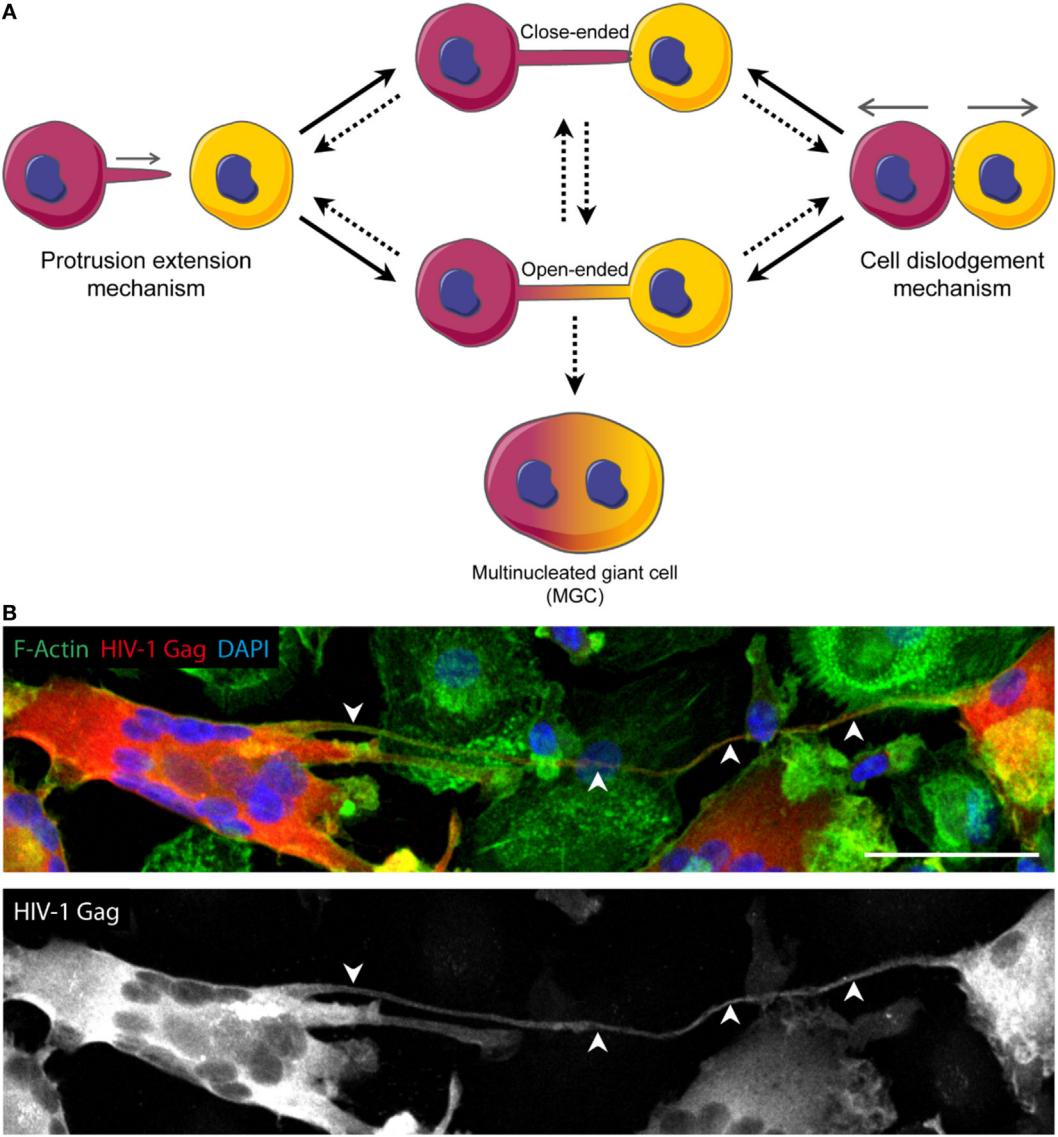
Models of tunneling nanotube (TNT) formation and putative role in the generation of multinucleated giant cells (MGC). **(A)** TNT can form according to two mechanisms: the “protrusion elongation” mechanism where the cell extends filopodia-like protrusion toward a specific target cell (left), and the “cell dislodgement” mechanism for which two cells initially in contact separate from each other, stuck by a thread of membrane that gives rise to a TNT (right). Each of these mechanisms can lead to either close-ended or open-ended TNT, the last one allowing cytoplasmic continuity between interconnected cells. The dynamics of close-ended and open-ended TNT formation is still not understood. In addition, TNT could either disconnect cells and thus abrogate their communication or could lead to MGC. **(B)** Confocal image of day 13 HIV-1-infected human monocyte-derived macrophages and MGC interconnected through a TNT. Arrowheads show a TNT. HIV-1 Gag (red), F-actin (green), DAPI (blue). Scale bar, 50 µm.

The main obstacle in reviewing the emerging TNT field is the different names given to these structures: TNT, cellular and membrane nanotubes, filopodia bridges, conduits or tubes, and nanotubules. Also, the huge number of publications on carbon nanotubes impedes the track of developments on TNT. Unifying terminology for nanotubes would thus be beneficial. In this mini-review, the term TNT will be used as done previously (2, 4). TNT are membranous channels connecting two or more cells over short to long distances. Actually, these structures can extend up to 200 µm in length in macrophages (5). To define TNT, we adopted the three phenotypic criteria proposed in a recent elegant review: (i) they connect at least two cells, (ii) they contain F-actin, and (iii) they do not touch the substrate (2). This definition allows the discrimination of TNT with any other F-actin-rich structures, such as filopodia. Regarding their functional properties, TNT transfer cytoplasmic molecules from one cell to another such as calcium, proteins or miRNA, mitochondria, several vesicles (e.g., lysosomes), pathogens, and cell-surface molecules; this ability constitutes the main functional criterion for TNT definition (6). The end of the structure can form a junctional border with the targeted cell (close-ended TNT) or the cytoplasm of the two connected cells can be mixed (open-ended TNT). On the one hand, the transfer of large molecules such as the lipophilic dye DiO is used to identify open-ended TNT (7). On the other hand, close-ended TNT form a junction at their end which are visualized by scanning electron microscopy (8). To avoid the past arguments on the need of cytoplasmic interactions for TNT, we shall consider in this review both close-ended and open-ended TNT (Figure 1A). As close-ended TNT mediate signal transfer through distant gap junctions (8, 9), they meet the functional criterion to be considered as TNT. Also, close-ended TNT could represent an intermediary status in the process of open-ended TNT formation. Finally, the group of Davis demonstrated that one particularity of macrophages is their ability to form different classes of TNT: thin ones (<0.7 μm in diameter), containing only F-actin; and thick ones (>0.7 μm), containing F-actin and microtubules (7). These two types of TNT could have different functions, as large material (e.g., lysosomes, mitochondria) can only travel between macrophages *via* thick TNT on microtubules (7).

## Discovery of TNT

The first description of functional TNT *in vitro* was made in rat kidney cells (PC12 cells) and human cell lines (10), followed immediately by the identification of similar structures in human monocytes and macrophages (11). It is now clear that TNT can form in several cell types, including cancer cells and most leukocytes. However, to our knowledge, TNT were not described in granulocytes. In DC, TNT appeared to be similar to those made by monocytes-derived macrophages (6, 12). However, unlike DC exposed to anti-inflammatory conditions, only those activated by pro-inflammatory conditions form complex network of TNT able to transfer soluble molecules and pathogens (13). Likewise, macrophages undergo different activation programs within the broad spectrum of pro- (M1) and anti-inflammatory (M2) polarization. Yet, their activation state has not been linked to the formation of TNT. The only available data concern the early HIV-1 infection of macrophages, driving them toward M1 polarization (14) and inducing a significant increase in TNT formation (5, 15–18).

While the majority of studies in TNT biology has been performed in one cell type (homotypic TNT) at a time, TNT formation between different cell types (heterotypic TNT) is not rare. In fact, TNT frequently form between macrophages or DC with another cell type, enabling the exchange of lysosomes, mitochondria, or viral proteins (16, 19–21).

The reason why TNT were discovered quite recently could be attributed to their fragility. Indeed, they are poorly resistant to the existing shearing forces in culture media, as well as light exposure and classical fixation methods. Thus, an appropriate way of performing live imaging is necessary to study TNT. When working on fixed cells, gentle fixation (e.g., glutaraldehyde-based fixation) should help preserve these highly delicate structures (22, 23).

## Formation of TNT

### Mechanisms of Formation

Cell examination by time-lapse microscopy suggested two mechanisms of TNT formation could exist. The first one proposes that two cells initially in contact separate from each other, remaining connected through a thin thread of membrane, which will be elongated upon cell separation (Figure 1A, right). The second puts forward that a cell would first bulge filopodia and extend them until reaching a neighboring cell, then converting towards TNT after making contact (24, 25) (Figure 1A, left). While the former is the prevailing mechanism in lymphoid cells, the latter one is observed in DC as TNT were reported to develop mainly from conversion of their filopodia (13, 19). In the case of macrophages, while they can use both mechanisms (6), the murine macrophage cell line (RAW 264.7 cells) mainly forms TNT from actin-driven protrusions, also called TNT-precursors (26). Of note, these two processes are not necessarily exclusive and could both occur between a given pair of cells. In either case, the requirement of F-actin is not questioned since treatment with latrunculin or cytochalasin D is often used to abolish TNT formation (2, 27, 28).

Regarding the opening of the conduit, and the potential transition between close-ended and open-ended TNT (Figure 1A), there is no proposed mechanism available. It is likely that the formation of open-ended TNT involves a step similar to what occurs during virus-to-cell membrane fusion or cell-to-cell fusion (29, 30), eventually leading to the generation of multinucleated giant cells (MGC) (Figure 1A).

### Molecular Actors

Few data are available to describe TNT at the molecular level. M-Sec, also known as tumor necrosis factor-α-induced protein, is one of the best characterized protein involved in TNT formation in macrophages. Its depletion in Raw264.7 cells reduces the formation of *de novo* TNT and their associated function (transfer of calcium flux) (22). Using the same macrophage cell line, the group of D. Cox recently showed that actin polymerization factors including the Rho GTPases family Rac1 and Cdc42, and their downstream effectors WAVE and WASP, participate in TNT formation (26). In addition, functional TNT are induced by the expression of the leukocyte specific transcript 1 (LST1) protein in HeLa and HEK cell lines. LST1 recruits the actin cross-linking protein filamin and the small GTPase RalA to the plasma membrane where it promotes RalA interaction with the exocyst complex, M-Sec, and myosin; these interactions trigger TNT formation (22, 23). Whether the mechanisms that operate in cell lines derived often from tumor origin apply to primary cells remains to be confirmed.

## *In Vivo* Relevance of TNT

A remaining question is to determine to what extent the *in vitro* data available in the literature are relevant *in vivo*. One of the problems is to apply *in vivo* the criteria of *bona fide* TNT (see above), in particular the requirement not to touch the substrate, which seems unlikely in 3D environments. In addition, testing the functionality of TNT in the context of tissues is challenging. Therefore, the structures observed *in vivo* should be carefully indicated as “TNT-like structures.” Key evidence for TNT-like structures *in vivo* comes from the immunology field providing the first images of thick TNT connecting DC in inflamed mouse corneas (31). To our knowledge, macrophage TNT have not been observed *in vivo* yet. The identification of specific molecular markers for TNT would be a great tool to confirm the existence of these structures *in vivo*. M-Sec, which is involved in TNT formation, cannot be considered as a specific marker since this ubiquitous protein is expressed all over the cytoplasm (5, 18, 28, 32, 33). Thus, one of the priority to progress in the TNT field is to characterize markers allowing unambiguous identification of cell-to-cell tubular connections as TNT.

## Role of TNT in Physiological Contexts

One of the most studied functions of TNT is the propagation of calcium flux. Calcium signaling through TNT helps regulate cell metabolism and communication between neurons (34). Interestingly, DC present the ability to establish calcium fluxes *via* TNT transmitted within seconds to other DC as far as 500 µm away from the donor cell (12). When TNT are disturbed by M-Sec knockdown, this calcium flux is inhibited (12, 22). DC have also the particularity to form TNT networks allowing the intercellular exchange of antigens (13), including in the context of MHC molecules as described between Hela cells (19, 27). Therefore, TNT could contribute to a higher efficiency in the antigen presentation process to activate adaptive immunity (19).

Another physiological role for TNT concerns the differentiation of osteoclasts (5, 18, 28, 32, 33). Osteoclasts are MGC derived from a myeloid precursor that present the unique ability to degrade the bone matrix, and thus to regulate bone homeostasis. Inhibition of TNT either by latrunculin B or by M-Sec depletion significantly suppresses osteoclastogenesis, and the M-Sec expression level increases during osteoclastogenesis (28, 35). Dendritic cell-specific transmembrane protein, a receptor involved in cell-to-cell fusion, has been shown to be transferred *via* TNT. The authors proposed that this process could participate in cell fusion among osteoclast precursors (28, 35). Moreover, nuclei are found inside large TNT-like structures (36), inferring that they participate in cell-cell fusion to generate OC. Elucidating the role of TNT in differentiation of MGC such as placental trophoblast, myotubes, and osteoclasts could be a new research area.

## Role of TNT in Pathological Contexts

Tunneling nanotubes not only contribute to cell-to-cell communication in physiological conditions but also in pathological processes. For example, the transfer of lysosomes from macrophages to fibroblasts, and of mitochondria from mesenchymal stromal cells to macrophages, are mediated by TNT and have important consequences in cystinosis and acute respiratory distress syndrome, respectively (20, 21).

Without the shadow of doubt, the most studied consequence of TNT in diseases is the transfer of pathogens, including prions, bacteria, and viruses [for review, see Ref. (1)]. One of the well-known example concerns the role of TNT in neurological diseases, especially when caused by prions (34). Actually, in addition to the TNT-dependent transfer of the infectious form of the prion protein (PrP^Sc^) between neuronal cells, TNT support PrP^Sc^ transfer from DC to the neurons in which PrP^Sc^ is further synthetized and transferred to the rest of the central nervous system (37). Regarding bacteria and viruses, some publications propose that they “surf” along TNT to spread from one cell to another (7, 13, 38–41). For example, in macrophages, live experiments show that *Mycobacterium bovis* bacillus Calmette–Guerin can travel along the surface of thin TNT, toward another macrophage, which will ingest it (7).

Viruses, including HIV-1, are well known to hijack the cytoskeleton in order to enter and travel inside their host cell, as well as towards bystander neighbor cells (5, 33, 39, 41, 42). For example, HIV-1 can actively induce the generation of filopodia in DC to propel virus particles towards neighboring cells. As one of the mechanism of TNT formation starts with membrane extension, filopodia formed upon HIV-1 infection could lead to TNT formation (2), especially in DC that develop networks of TNT from elongation of their dendrites (13, 19). Importantly, the formation of TNT by DC favors trans-infection of targeted CD4^+^ T lymphocytes at a relatively long distance, similar to what happens between two distant CD4^+^ T lymphocytes (8).

In macrophages, HIV-1 induces TNT formation and potentially uses them to spread (18). Whether thin or thick TNT are formed is unknown. Assuming that thick TNT are induced, HIV-1 could travel inside these structures by using a microtubule-dependent movement, in addition to the described “surfing” of HIV-1 at the surface of TNT. Despite the fact that Gag and Nef proteins and HIV-1-containing vesicles have been detected inside TNT, there are no convincing experiments in living cells available to prove that HIV-1 travels inside TNT and infects the targeted cell (5, 15, 17, 18). Pushing live imaging to super-resolution microscopy techniques would be of great help to study how HIV-1 traffics using TNT.

In light of the importance of macrophages in HIV-1 pathogenesis (43–45), it is crucial to bridge the several gaps that blur our understanding of the role of TNT in macrophages during HIV-1 infection. First, it is important to determine whether HIV-1-induced TNT in macrophages are close- or open-ended to better understand how HIV-1 traffics *via* TNT. Second, whether TNT from a HIV-infected cell could target non-infected cells remains to be elucidated. It would be an efficient way for the virus to spread around without being detected. Finally, the molecular regulation of HIV-1-induced TNT in macrophages has only started to be elucidated. The HIV-1 Nef protein could play a central role in TNT formation by interacting with members of the exocyst complex (16, 18, 46, 47). Moreover, Nef modulates F-actin and cell migration (48), two effects which could participate in TNT generation. Finally, a hallmark of HIV-1 infection is the formation of MGC, a process that can be driven by TNT in order to persist during late infection stages, when most infected macrophages are MGC (Figure 1B) (32, 33). Interestingly, both HIV-1-induced TNT and MGC are reduced when macrophages are infected with *nef*-deleted viruses (18, 32, 33).

Importantly, while TNT spread the virus among HIV-1 target cells (T lymphocytes, macrophages, and DC), TNT also affects the nature of infection by circumventing the need for classical receptor-mediated virus entry or transfer viral components to cells that are not susceptible to infection. As a matter of fact, the transfer of Nef *via* TNT between infected macrophages and B cells induces drastic B cell abnormalities at the systemic and mucosal level (16).

## Conclusion

The TNT field requires the unification of the terminology and definition of TNT, as well as the development of new tools adapted for the detection and monitoring of these particular structures. The main challenge so far is to discover molecular markers to specifically identify TNT, especially *in vivo*. To this end, an automated siRNA-based screen could be used in *in vitro* conditions for which TNT formation is controlled, as performed for the virological synapse (49). Another issue is the fragility of TNT which complicates their manipulation. Thus, the use of specific experimental conditions or devices, such as microfluidic systems (50), is needed. Moreover, it would be helpful to study the opening of close-ended TNT in terms of molecular components and dynamics. Likewise, it is imperative to determine whether TNT formation and regulation can be influenced by extracellular stimulti and/or tissue microenvironment in pertinent *in vivo* physiological and pathological contexts. For example, during HIV-1 infection, TNT represent a new way for viral spread. However, the literature remains scarce, rising far more questions than answers. Interestingly, HIV-1 and other microbes can serve as efficient tools to better understand TNT structure and function. Furthermore, TNT-based studies in the HIV-1 field are needed to better understand viral dissemination and pathogenesis. The particularity of TNT to perform “intimate” communication with a specific partner is probably key in HIV-1 spread. A tempting hypothesis is that infected cells could direct their TNT towards uninfected cells. This way, the virus could spread without being detected by the surveilling immune system. Finally, new insights into the mechanisms of TNT formation and regulation would be of high relevance to design novel therapeutics for several diseases, including viral infections.

## Author Contributions

MD, SS, IM-P, and CV wrote the manuscript. SS created the figure. GL-V edited the manuscript.

## Conflict of Interest Statement

The authors declare that the research was conducted in the absence of any commercial or financial relationships that could be construed as a potential conflict of interest.
